# Supplementary material for: A Case Report of a Patient on Therapeutic Warfarin Who Died of COVID-19 Infection with a Sudden Rise in D-Dimer
Source: Biomedicines. 2021 Oct 3;9(10):1382. doi: 10.3390/biomedicines9101382 (PMC8533641; doi:10.3390/biomedicines9101382)
Supplement: Supplementary file 1 [file biomedicines-09-01382-s001.zip › biomedicines-1378927-supplementary.pdf]

Supplementary Tables

Table S1. Past medical history.

**Past Medical History**
Hypertension
Hyperlipidemia
Mild Coronary Artery disease managed by lifestyle changes 2017
Cardiomyopathy 2017 (EF <40%) Managed by lifestyle changes
Diabetes hga1c 6.2
h/o bilateral PE (Pulmonary embolism) provoked DVT (injury) 2013
Recurrent saddle PE 2015 on lifetime warfarin
Provoked DVT from injury 2013
Anemia, low folate
Intermittent elevated LFT-fatty liver
Pelvo-abdominal horse-shoe kidney
Hep B immune from exposure
Osteoarthritis spondylolisthesis spine
Left leg injury from tree 2013
Benign Prostate Hyperplasia
Mild Chronic Obstructive Pulmonary Disease
Low vitamin D
**Social History**
Tobacco remission
Alcohol intermittent
Drugs Denied

PE: Pulmonary embolism; DVT: Deep Vein Thrombosis; LFT Liver function Tests; Hep B: Hepatitis B.

Table S2. Home medication list.

	**List Of Medicines at Home**	**Status**
1.	ACCU-Chek guide (glucose) Test strip use: 1 Strip for testing as directed to test the blood glucose	
2.	Accu-chek guide(glucose) Hi/Lo control solution: 1 drop directed to test Accu-Chek guide machine	
3.	Atenolol 100 mg tab; take one tablet by mouth every morning for blood pressure. *new dose*	
4.	Insulin, glargine, human solostar 3ml pen inject 22 units under the skin at bedtime discard pen 28 days after opening.	
5.	Losartan 100 mg tab take one tablet by mouth every day for blood pressure.	
6.	Needle, pen 31 g, 5 mm use one needle under the skin each day. Use with insulin pen.	
7.	Rosuvastatin CA 40 mg tab take one-half tablet by mouth every day for cholesterol replacement atorvastatin.	
8.	Warfarin NA (Teva) 5 mg tab; take one tablet by mouth on Monday, Wednesday, and Friday and take one and one-half tablets all other days. Take at the same time every day to prevent blood clotting.	
9.	Terazosin 4 mg QHS	
10.	Dulaglutide 1.5 mg/0.5 ml injection pen<br>Inject 1.5 mg/0.5 ml under the skin once a week<br>Rx# 9289630 last released: 8/26/20, qty/days supply: 12/84<br>Rx expiration date: 12/5/20, refills remaining: 0	
11.	Hydrochlorothiazide 25mg tab<br>Take one tablet by mouth every morning for blood pressure<br>Rx# 9197923a last released: 8/27/20, qty/days supply: 90/90<br>Rx expiration date: 9/24/20, refills remaining: 1	
12.	Insulin, Glargine, Human solostar 3 ml pen<br>Inject 22 units under the skin at bedtime discard pen 28 days after opening<br>Rx# 9162481a last released: 9/30/20, qty/days supply: 5/60<br>Rx expiration date: 8/25/21, refills remaining: 4	
13.	MVI OTC	

Table S3. Vitals signs in Emergency department.

Signs	
Temperature	97.7–98-F
Heart Rate	87–88
Blood pressure	201/110–140/92
Respiration rate	14–18
O_2 at rest on arrival	91–93% Room Air
Vitals on minimal exertion standing & few steps	120, RR 22-24, O2 sat 86%, BP 96/56
Oxygen saturation	2-3 L, 93–96% at rest in bed

Table S4. Inpatient medication list.

	Active inpatient medications	Status
1.	Aerochamber Chamber One aerochamber INHL BID for use for budesonide/formoterol	Active
2.	Ascorbic Acid tab 500 mg PO TID	Active
3.	Atenolol tab 100 mg PO QAM	Active
4.	Budesonide/formoterol INHL, Oral 2 Puffs INHL Oral BID with spacer	Active
5.	Ceftriaxone injection, soln 2 gm/1 vial IVP qday mix with 20 ml ns and give ivp over 3–5min 20 ml ns and give ivp over 3–5 min	Active
6.	Cholecalciferol tab 100 mcg po qday	Active
7.	D-50-W Injection, Soln 50cc IV Q1H PRN. Give for the patient with glucose <70 and showing signs of severe hypoglycemia. In over 5–15 minutes.	Active
8.	Dextrose 40% (glucose) gel, oral 1 tube po q1h prn Glucose < 70 and signs of mild hypoglycemia (sweating, hunger, tachycardia) then give one tube = 31 g (24 gs carbohydrates) Glucose gel	Active
9.	Dextrose 40% (glucose) gel, oral 2 tubes po q1h prn. Glucose < 70 and signs of moderate hypoglycemia (disorientation, confusion, hostility) then give two tubes = 62 g (48 g carbohydrates) Glucose gel	Active
10.	Doxycycline injection, Doxycycline 100 mg in normal saline 100 ml. Infuse over 60 min ivpb Q12 h	Active
11.	Enoxaparin injection 90 mg/0.9 ml sq q12 h	Active
12.	Folic acid tab 2 mg po qday	Active
13.	Glucagon injection 1mg/1 vial IM Q1H PRN. For glucose <70 and patients with signs of severe hypoglycemia; May be given IM, SQ, or IV.	Active
14.	Insulin human regular injection per sliding scale sq q1 h prn pre-meal bg 150–199 = 1 Unit, 200–249 = 2 Units, 250–299 = 3 Units, 300–349 = 4 Units, >349 = 6 Units and Call MD	Active
15.	Insulin glargine human injection 35 units sq qhs. Notify MD if bg < 100 or pt changed to NPO status	Active
16.	Lactulose solution, Oral 30 ml PO BID hold if >1 BM	Active
17.	Losartan tab 100 mg PO QDAY hold if SBP < 100	Active
18.	Melatonin cap/tab 9 mg PO QHS	Active
19.	Message to nursing miscellaneous Accu check by nursing misc achs	Active
20.	Methylprednisolone inj, soln methylprednisolone 80 mg in NS 250 ML, 10 ml/h@0 Begin after bolus IV.	Active
21.	Pantoprazole Tab, EC 40 mg PO ACB	Active
22.	Potassium phosphate injection, solution Potassium phosphate 15 MM in D5W 100 ML infuse over 120 min 2 doses for total fo 30mm IVPB Q2H	Active
23.	Remdesivir (lyophilized) inj remdesivir (lyophilized) 100 mg in NS 250 ml infuse over 60 min for five days starting tomorrow IV Qday	Active
24.	Rosuvastatin tab 20 mg PO Qday	Active
25.	Senna tab 17.2 mg PO HS	Active
26.	Terazosin cap,oral 4 mg PO QPM	Active
27.	Thiamine tab 100 mg PO Qday	Active
28.	Zinc sulfate cap, oral 50 mg PO Qday	Active
